# The efficacy of an integrated empowerment and narrative nursing program on enhancing professional identity and alleviating burnout among neonatal nurses: a cluster randomized controlled trial

**DOI:** 10.3389/fpubh.2025.1745441

**Published:** 2026-01-20

**Authors:** Sudan Liu, Zhengli Kang, Huimin Li, Yaqing Du, Junran Li, Zeyang Zhang

**Affiliations:** 1 Neonatal Department, Shijiazhuang Fourth Hospital, Shijiazhuang, Hebei, China; 2 Department of Obstetrics, Shijiazhuang Fourth Hospital, Shijiazhuang, Hebei, China; 3 Department of Operating Room, Shijiazhuang Fourth Hospital, Shijiazhuang, Hebei, China

**Keywords:** empowerment intervention, narrative nursing, neonatal nurses, nurse burnout, professional identity

## Abstract

**Background:**

Neonatal nurses frequently report lower professional identity and high levels of burnout owing to heavy workloads, emotional demands, and staffing pressures. This study evaluated an integrated empowerment and narrative nursing (I-ENP) program designed to enhance professional identity and reduce burnout among neonatal nurses.

**Methods:**

We conducted a two-arm, parallel, cluster randomized controlled trial in four tertiary hospitals in Hebei Province, China, from January to October 2023. Hospital clusters were randomized to I-ENP or control (usual care). The I-ENP group received a structured 6-month program comprising four empowerment education modules and six narrative nursing sessions; the control group received no additional intervention. The primary outcome was change in the Professional Identity Scale for Nurses (PISN) from baseline to 6 months. Secondary outcomes were changes in the Maslach Burnout Inventory (MBI) subscales (emotional exhaustion, depersonalization, personal accomplishment) and nurse turnover intention, measured at baseline, 6 months (end of intervention), and 9 months (3-month follow-up).

**Results:**

One hundred seventy-two neonatal nurses completed the study (85 I-ENP, 87 control). At 6 months, the I-ENP group demonstrated greater improvement in PISN compared with control (mean difference 15.8 points; 95% CI: 12.5–19.1; *P* < 0.001). Emotional exhaustion and depersonalization declined significantly [mean differences −8.2 (95% CI: −10.5 to −5.9) and −4.1 (95% CI: −5.7 to −2.5), respectively; both *P* < 0.001], while personal accomplishment increased (mean difference 6.5; 95% CI: 4.8–8.2; *P* < 0.001). These benefits were largely sustained at 9 months, and turnover intention was lower in the I-ENP group than control (11.8 vs. 29.9%; *P* = 0.003).

**Conclusions:**

This cluster randomized trial provides promising evidence that an integrated empowerment and narrative nursing program may be effective in enhancing professional identity and alleviating burnout among neonatal nurses, suggesting it is a potentially useful intervention strategy to support nurse wellbeing. The findings warrant further evaluation in diverse hospital settings and populations to determine broader applicability and sustainability.

## 1 Introduction

Neonatal nursing is a highly specialized and demanding field, requiring advanced clinical skills, constant vigilance, and profound emotional resilience (1, 2). Nurses in Neonatal Intensive Care Units (NICUs) face unique stressors, including the high mortality and morbidity of their patients, frequent ethical dilemmas, and emotionally taxing interactions with distressed families (3, 4). These chronic stressors place neonatal nurses at a high risk for developing professional burnout, a syndrome characterized by emotional exhaustion, depersonalization, and a reduced sense of personal accomplishment (5). High burnout rates are strongly linked to decreased job satisfaction, higher turnover intention, and compromised patient safety (6, 7).

A critical factor intertwined with burnout is professional identity, defined as an individual's sense of “being a nurse,” which is shaped by professional values, beliefs, and experiences (8). A strong professional identity serves as an internal motivator, fostering commitment, job satisfaction, and resilience against workplace adversity (9, 10). Conversely, a weak professional identity can exacerbate burnout and contribute to workforce attrition, a significant challenge in a field already facing shortages (11, 12). Therefore, developing effective interventions that can simultaneously bolster professional identity and mitigate burnout is a strategic imperative for sustaining a stable and competent neonatal nursing workforce (9, 13).

Two promising psychological and educational approaches are empowerment education and narrative nursing. Empowerment education aims to enhance individuals' sense of control, self-efficacy, and problem-solving abilities by fostering skills in areas like stress management, communication, and team collaboration (14). Narrative nursing, rooted in narrative medicine, uses storytelling and reflective writing to help nurses process difficult experiences, find meaning in their work, and enhance empathy and humanistic care (15, 16). While both interventions have shown benefits in various nursing specialties (17, 18), their systematic integration and application specifically for neonatal nurses remain underexplored. The synergy of these two approaches could be particularly potent: empowerment education provides practical skills to manage external stressors, while narrative nursing offers a reflective space to process internal emotional challenges and reaffirm professional values.

This study reports the findings of a cluster randomized controlled trial designed to evaluate the efficacy of an Integrated Empowerment and Narrative Nursing Program (I-ENP) for neonatal nurses. We hypothesized that participants in the I-ENP group, compared to those receiving usual care, would demonstrate a significant improvement in professional identity and a significant reduction in burnout symptoms at the end of the 6-month intervention, with effects maintained at a 3-month follow-up.

## 2 Methods

### 2.1 Study design and setting

This study was a two-arm, parallel-group, cluster randomized controlled trial conducted across four large tertiary-level teaching hospitals in Shijiazhuang, Hebei Province, China. The unit of randomization (cluster) was the hospital to minimize contamination between the intervention and control groups. The study protocol was approved by the Institutional Review Board of our hospital. This report adheres to the Consolidated Standards of Reporting Trials (CONSORT) 2010 statement for cluster trials (19).

### 2.2 Participants and eligibility

Registered nurses working full-time (≥1 year of experience) in the NICUs of the participating hospitals were eligible. We excluded nurses who were on long-term leave (e.g., maternity leave), in managerial positions, or planning to leave their position within the study period. All participants provided written informed consent before enrollment.

### 2.3 Randomization and blinding

The four participating hospitals (clusters) were randomized in January 2023 in a 1:1 ratio to either the I-ENP group or the control group. An independent statistician, not involved in recruitment or data collection, performed the randomization using a computer-generated random number sequence. Nurses were recruited and consented to participate after cluster allocation, from January to February 2023. Although this post-allocation recruitment could theoretically allow for selection bias, we minimized this risk by: (a) using standardized, pre-defined eligibility criteria communicated equally to all four hospitals; (b) having recruitment conducted by nursing HR personnel blinded to the study hypothesis; and (c) performing detailed baseline demographic and clinical comparisons between groups, which showed no significant differences. Due to the nature of the educational intervention, it was not possible to blind the participants or the intervention facilitators. However, the data collectors and statisticians who analyzed the data were blinded to the group assignments.

### 2.4 Intervention

The I-ENP was a structured 6-month program designed based on empowerment theory and narrative practice principles (20, 21). The program was developed by a multidisciplinary team of nursing experts, psychologists, and neonatologists and was pilot-tested for feasibility and acceptability. Details of the program are provided in Supplementary Table 1.

The I-ENP consisted of two core components delivered in small groups (8–10 nurses per group) led by two trained facilitators (a senior nursing manager and a certified psychological counselor). Prior to delivering the intervention, facilitators completed a 2-day intensive training workshop that included: (a) overview of empowerment theory and narrative practice principles; (b) detailed review of each module and session outline; (c) practice delivery of sample sessions and feedback; (d) techniques for facilitating safe, non-judgmental group discussions; and (e) crisis response protocols for nurses in distress. A detailed facilitator manual was developed, including session-by-session scripts, timing guides, discussion prompts, and worksheets for nurses. Fidelity checks were performed by an independent observer who attended two sessions (one empowerment workshop and one narrative session) from each facilitator pair (*n* = 4 pairs), using a standardized checklist that assessed adherence to key content points, quality of group engagement, and adherence to timing. All observed sessions achieved ≥90% fidelity score, indicating high consistency with the protocol.

#### 2.4.1 Empowerment education modules

This component comprised 4 monthly workshops (2 h each). Module 1 (Professional Competence) covered advanced neonatal care techniques, case discussions of complex clinical scenarios, and individual goal-setting for professional development. Module 2 (Stress Management) included identification of workplace stressors, practice of mindfulness and relaxation techniques (e.g., progressive muscle relaxation, guided imagery), and building emotional regulation skills. Module 3 (Effective Communication) focused on communication strategies with distressed families, assertive communication with colleagues, conflict resolution techniques, and role-playing of difficult conversations (e.g., discussing poor prognosis with parents). Module 4 (Teamwork and Collaboration) addressed team dynamics, psychological safety, and structured team problem-solving exercises.

#### 2.4.2 Narrative nursing sessions

This component included six bi-weekly group sessions (1.5 h each) conducted over months 1–6 of the intervention. Sessions were structured around: (1) Story Sharing, where nurses shared memorable or challenging clinical experiences; (2) Reflective Writing, where participants wrote “nursing diaries” to document and explore their emotional responses and professional insights; and (3) Empathetic Listening and Peer Support, where guided discussions focused on finding shared meaning and providing mutual support. Among the 90 nurses allocated to the I-ENP group, 85 completed the study. Of these 85, the mean number of empowerment workshops attended was 3.8 out of 4 (SD = 0.4; range 3–4), and the mean number of narrative sessions attended was 5.7 out of 6 (SD = 0.5; range 5–6). Overall, 78 of 85 (91.8%) nurses attended all four empowerment modules, and 80 of 85 (94.1%) attended at least five of the six narrative sessions. For the 5 nurses lost to follow-up, they had attended a mean of 3.4 workshops and 4.8 narrative sessions before withdrawing. High attendance and adherence rates support the feasibility and acceptability of the intervention.

The control group received usual care, which included: (a) standard hospital-provided continuing nursing education, typically 1–2 h per month, focused on clinical skills updates and procedural competencies (e.g., ventilator management, infection control) with minimal or no attention to professional identity or burnout; (b) access to employee assistance programs (EAP) offered by the hospital, including telephone counseling and limited on-site mental health resources, but with low uptake (estimated <10% of nurses utilized EAP); and (c) routine departmental meetings and grand rounds. Notably, systematic interventions addressing professional identity, empowerment, or narrative reflection were not standard practice in these hospitals prior to the study.

### 2.5 Outcomes and measurements

Data were collected via secure, web-based self-report questionnaires at baseline (T0), 6 months (T1, post-intervention), and 9 months (T2, 3-month follow-up).

#### 2.5.1 Primary outcome

The primary outcome, pre-specified prior to analysis, was the change in the Professional Identity Scale for Nurses (PISN) score from baseline (T0) to 6 months (T1, end of intervention). This is measured by the Chinese version of the Professional Identity Scale for Nurses (PISN) (22). This 30-item scale assesses five dimensions: professional identity cognition, benefit perception, leadership, social modeling, and professional identity uniqueness. Items are rated on a five-point Likert scale, with total scores ranging from 30 to 150. Higher scores indicate a stronger professional identity. The scale has demonstrated good reliability and validity in Chinese nurses (Cronbach's α = 0.94) (23, 24).

#### 2.5.2 Secondary outcomes

Secondary outcomes, also pre-specified, were: (1) changes in Maslach Burnout Inventory subscale scores from baseline to 6 months and from baseline to 9 months; (2) change in turnover intention at 6 months; and (3) exploratory: PISN scores at 9-month follow-up.

#### 2.5.3 Burnout

Measured using the Chinese version of the Maslach Burnout Inventory-Human Services Survey (MBI-HSS) (25). This 22-item scale consists of three subscales: Emotional Exhaustion (EE, nine items), Depersonalization (DP, five items), and low Personal Accomplishment (PA, eight items). Higher scores on EE and DP and lower scores on PA indicate greater burnout. The scale has shown good psychometric properties (Cronbach's α > 0.70 for all subscales) (26, 27).

#### 2.5.4 Turnover intention

Measured at 6 months only, using a single item: “Are you seriously considering leaving your current position in the next 12 months?” with a “Yes/No” response. We did not measure turnover intention at 9 months because the question referred to intention to leave “in the next 12 months,” which would be retrospectively answered at 9 months and prone to recall bias.

Demographic and work-related information, including age, gender, education level, marital status, and years of nursing experience, was collected at baseline.

### 2.6 Statistical analysis

The sample size was calculated based on the primary outcome. Assuming a mean difference of 8 points in the PISN score between groups, a standard deviation of 15, a two-sided significance level (α) of 0.05, and a power (1–β) of 0.90, a sample of 75 participants per group was required. The intracluster correlation coefficient (ICC) was assumed to be 0.02, based on a review of cluster randomized trials in nursing and healthcare settings, where ICCs for organizational or educational interventions typically range from 0.01 to 0.05. This conservative mid-range value was selected to balance Type I and Type II error risks (Detailed observed ICCs for all outcomes are provided in Supplementary Table 2). The design effect (DE) was calculated as DE = 1 + (m – 1) × ICC, where m is the average cluster size. With m = 45 (average cluster size of ~90 nurses per hospital divided into roughly equal I-ENP and control groups) and ICC = 0.02, the DE = 1 + (45 – 1) × 0.02 = 1.88. This means that the effective sample size is 1.88 times smaller than the nominal sample size. Accounting for this, a nominal sample of 75 per group (total 150) was inflated to 141 per group to maintain 90% power, and a 15% margin was added for anticipated attrition, yielding a final target of at least 170 participants (85 per group). The actual attrition was 8 out of 180 (4.4%), well below the 15% anticipated, and the final analysis included 172 participants.

All analyses were performed on an intention-to-treat (ITT) basis, including all randomized participants. Missing data were handled using multiple imputation by chained equations (MICE). We generated *m* = 20 imputations, assuming data were missing at random (MAR). The imputation model included: (a) all outcome variables (PISN and MBI subscale scores at all three time points); (b) baseline covariates (age, gender, marital status, educational level, years of nursing experience); (c) group assignment (I-ENP vs. control); (d) hospital cluster identifier; and (e) auxiliary variables related to dropout patterns. Imputations were performed separately within each hospital cluster to preserve within-cluster correlations. Imputation methods used were predictive mean matching for continuous variables and logistic regression for binary variables.

To account for the clustered nature of the data and the small number of clusters (*m* = 4), linear mixed-effects models were used to compare the changes in continuous outcomes (PISN and MBI subscale scores) from baseline to 6 and 9 months, with group and time as fixed effects and hospital (cluster) as a random effect. We justified the use of individual-level mixed-effects models despite the small number of clusters because: (i) the hospitals were the unit of randomization, and accounting for their random variation is conceptually appropriate; (ii) permutation tests and exact methods are computationally intensive with our outcome structure; (iii) we performed cluster-level sensitivity analyses (see below). The model included baseline scores as a covariate to increase statistical power. In addition to the individual-level mixed-effects model, we performed cluster-level summary analyses as a sensitivity check. We re-analyzed the primary outcome (PISN) using a cluster-level independent samples *t*-test, comparing the mean change from baseline to 6 months for the two intervention clusters vs. the two control clusters. We also performed bootstrap resampling (1,000 iterations at the cluster level) to obtain confidence intervals (see Supplementary Table 3 for full sensitivity analysis results).

Categorical outcomes (turnover intention) were analyzed using a generalized estimating equation (GEE) model. A *P*-value < 0.05 was considered statistically significant. All analyses were conducted using R version 4.2.2.

## 3 Results

### 3.1 Participant flow and baseline characteristics

**Figure 1:**
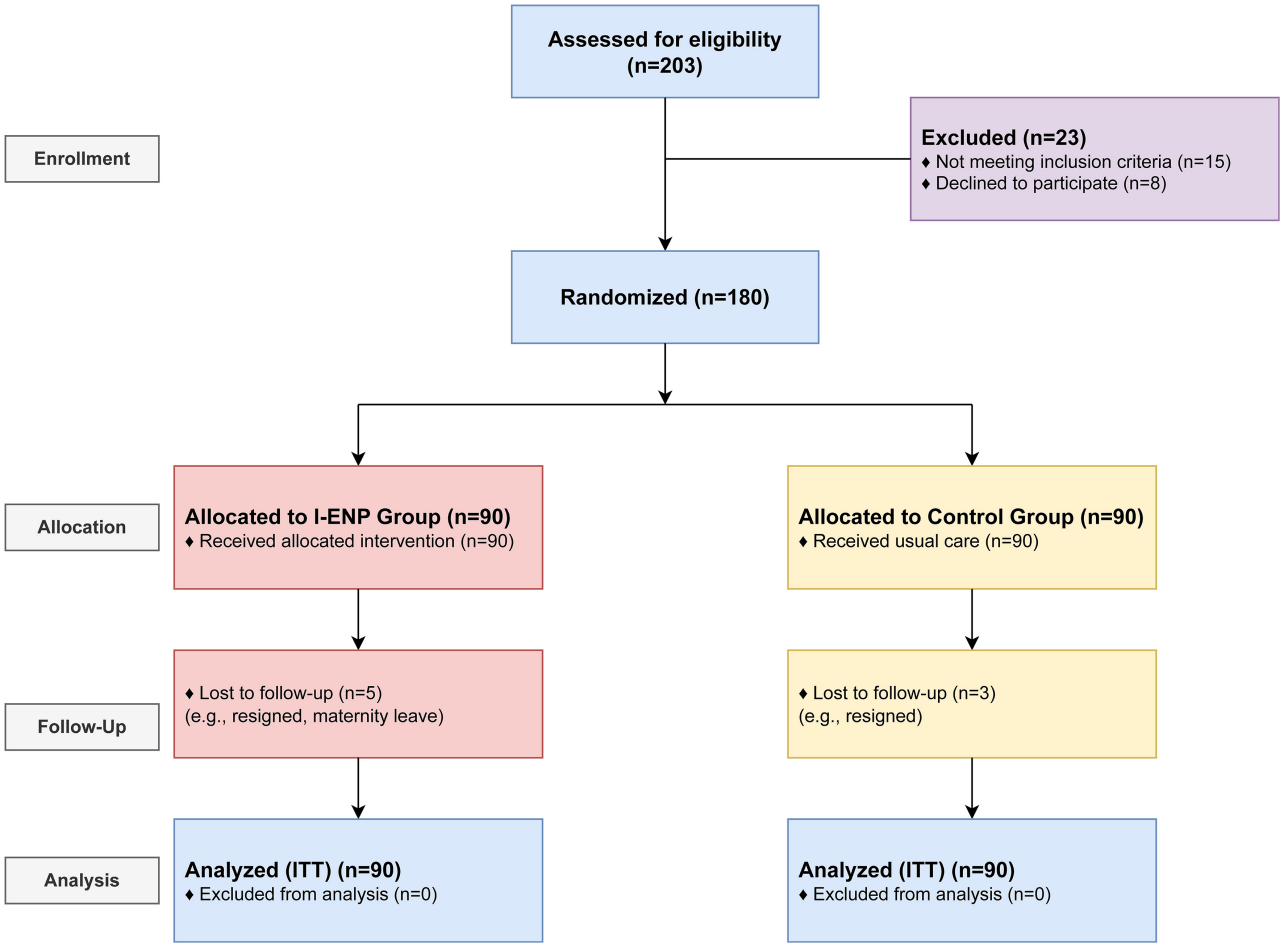
CONSORT 2010 flow diagram. The diagram illustrates the flow of participants (hospitals as clusters and individual nurses) through the enrollment, allocation, follow-up, and analysis phases of the cluster randomized controlled trial.

From January to February 2023, 203 nurses were assessed for eligibility across the four hospitals. A total of 180 nurses met the inclusion criteria and consented to participate. The four hospitals were randomized, with two hospitals (*n* = 90 nurses) allocated to the I-ENP group and two hospitals (*n* = 90 nurses) to the control group. During the 9-month study period, five nurses from the intervention group and three from the control group were lost to follow-up. Finally, 172 participants (85 in the I-ENP group, 87 in the control group) were included in the ITT analysis. The participant flow is detailed in the CONSORT diagram (Figure 1).

Baseline demographic and professional characteristics were well-balanced between the two groups (Table 1). The mean age of participants was 28.5 (SD = 4.2) years, and the majority were female (97.1%). The average years of experience in the NICU was 5.6 (SD = 3.1) years. There were no significant differences in baseline scores for professional identity or burnout subscales between the two groups.

**Table 1:** Baseline demographic and professional characteristics of participants.

**Characteristic**	**I-ENP group (*n* = 85)**	**Control group (*n* = 87)**	***P*-value**
Age (years), mean (SD)	28.3 (4.1)	28.7 (4.3)	0.58^a^
**Gender**, ***n*** **(%)**			
Female	82 (96.5)	85 (97.7)	0.99^b^
Male	3 (3.5)	2 (2.3)	
**Marital status**, ***n*** **(%)**			
Unmarried	48 (56.5)	51 (58.6)	0.75^b^
Married	37 (43.5)	36 (41.4)	
**Educational level**, ***n*** **(%)**			
Bachelor's degree or above	65 (76.5)	68 (78.2)	0.81^b^
Below Bachelor's degree	20 (23.5)	19 (21.8)	
Years in nursing, mean (SD)	6.1 (3.5)	6.4 (3.8)	0.62^a^
Years in NICU, mean (SD)	5.5 (3.0)	5.7 (3.2)	0.71^a^
**Baseline outcome scores, mean (SD)**			
Professional identity (PISN)	105.2 (14.1)	104.8 (13.5)	0.84^a^
Emotional exhaustion (MBI-EE)	28.5 (6.8)	29.1 (7.2)	0.59^a^
Depersonalization (MBI-DP)	11.2 (3.5)	11.5 (3.8)	0.61^a^
Personal accomplishment (MBI-PA)	32.8 (5.5)	32.1 (5.9)	0.45^a^

Data are presented as mean (standard deviation) or n (%). I-ENP, integrated empowerment and narrative program; NICU, neonatal intensive care unit; PISN, professional identity scale for nurses; MBI, Maslach burnout inventory.

a P-value calculated using independent samples t-test.

b P-value calculated using Chi-square test or Fisher's exact test.

### 3.2 Primary outcome: professional identity

**Figure 2:**
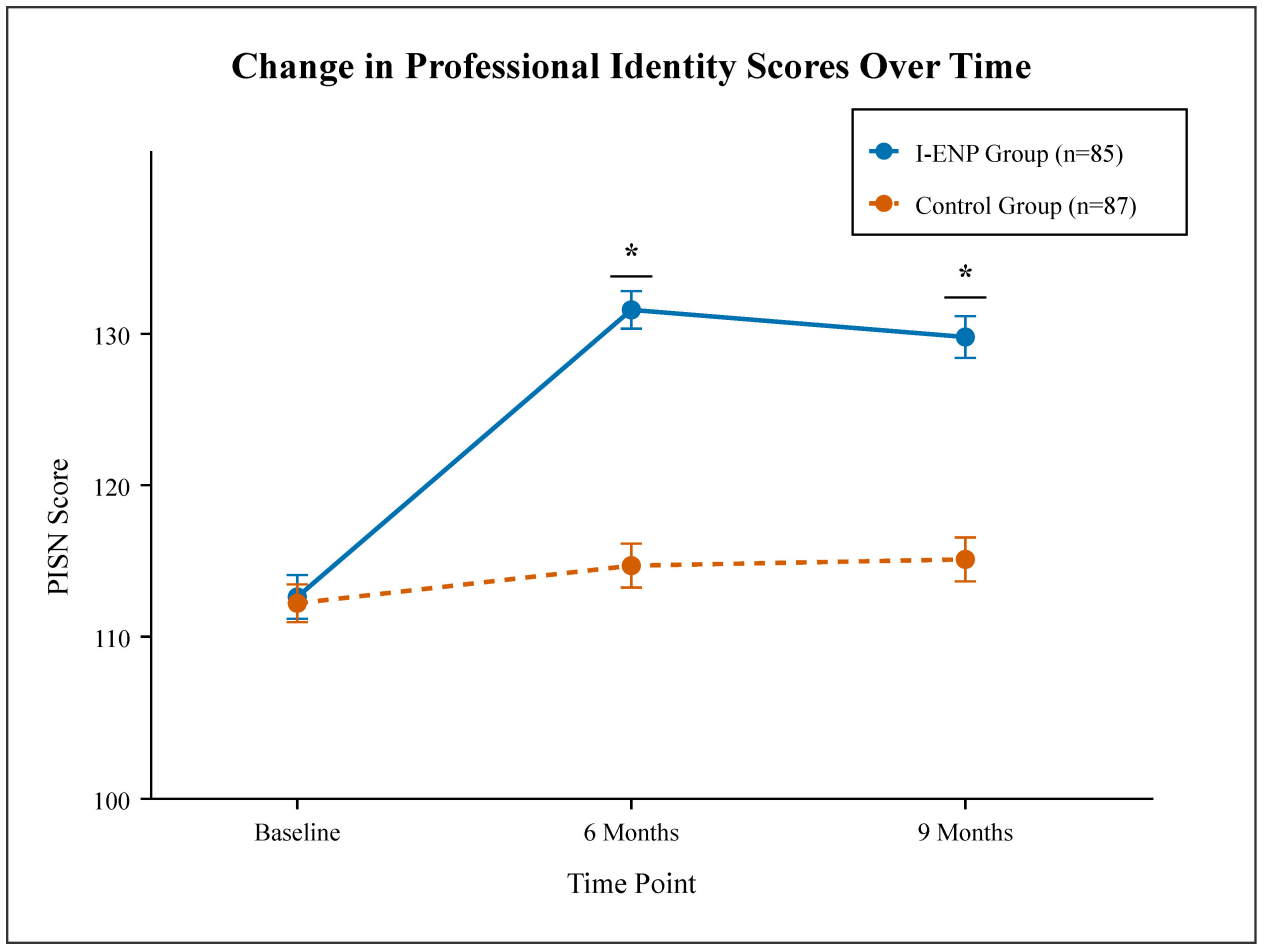
Change in professional identity scores (PISN) over time. Mean scores for the integrated empowerment and narrative program (I-ENP) group and control group at baseline, 6 and 9 months. Error bars represent 95% confidence intervals. *P*-values were calculated using linear mixed-effects models adjusted for clustering and baseline scores *P < 0.001.

The results of the linear mixed-effects model are presented in Table 2. At 6 months, the I-ENP group had a significantly greater increase in the PISN score compared to the control group. The mean PISN score in the I-ENP group increased from 105.2 (SD = 14.1) at baseline to 123.5 (SD = 11.8) at 6 months, while the control group's score changed from 104.8 (SD = 13.5) to 107.2 (SD = 13.9). The adjusted mean difference between the groups at 6 months was 15.8 points (95% CI: 12.5–19.1; *P* < 0.001). This significant effect was sustained at the 9-month follow-up, with an adjusted mean difference of 13.5 points (95% CI: 10.1–16.9; *P* < 0.001; Figure 2).

**Table 2:** Primary and secondary outcomes at 6 and 9 months.

**Outcome**	**Group**	**Baseline (T0) mean (SD)**	**6 months (T1) mean (SD)**	**9 months (T2) mean (SD)**	**Adjusted mean difference (95% CI) at T1^a^**	***P*-value (T1)**	**Adjusted mean difference (95% CI) at T2^a^**	***P*-value (T2)**
Professional identity (PISN)	I-ENP (*n* = 85)	105.2 (14.1)	123.5 (11.8)	121.8 (12.5)	15.8 (12.5 to 19.1)	<0.001	13.5 (10.1 to 16.9)	<0.001
	Control (*n* = 87)	104.8 (13.5)	107.2 (13.9)	107.6 (14.1)				
Emotional exhaustion (MBI-EE)	I-ENP (*n* = 85)	28.5 (6.8)	19.8 (5.5)	21.1 (5.9)	−8.2 (−10.5 to −5.9)	<0.001	−7.0 (−9.4 to −4.6)	<0.001
	Control (*n* = 87)	29.1 (7.2)	28.6 (7.0)	28.8 (7.1)				
Depersonalization (MBI-DP)	I-ENP (*n* = 85)	11.2 (3.5)	6.9 (2.8)	7.5 (3.1)	−4.1 (−5.7 to −2.5)	<0.001	−3.3 (−4.9 to −1.7)	<0.001
	Control (*n* = 87)	11.5 (3.8)	11.3 (3.7)	11.2 (3.9)				
Personal accomplishment (MBI-PA)	I-ENP (*n* = 85)	32.8 (5.5)	39.9 (4.8)	38.5 (5.1)	6.5 (4.8 to 8.2)	<0.001	5.6 (3.8 to 7.4)	<0.001
	Control (*n* = 87)	32.1 (5.9)	32.7 (6.0)	32.3 (6.2)				

CI, confidence interval; SD, standard deviation; I-ENP, integrated empowerment and narrative program.

a Adjusted mean difference between groups at 6 months (T1) and 9 months (T2), calculated using a linear mixed-effects model adjusted for baseline scores and clustering by hospital with Kenward-Roger approximation for degrees of freedom.

### 3.3 Sensitivity analyses

In the cluster-level independent samples *t*-test comparing the two intervention clusters to the two control clusters, the results (mean difference = 15.9 points; 95% CI: 11.3–17.1; *P* = 0.002) were concordant with the individual-level analysis (Supplementary Table 3). Bootstrap resampling (1,000 iterations at the cluster level) yielded a 95% bootstrap CI of [11.8, 17.6] for the intervention effect on PISN, again supporting the main conclusion. The observed ICCs for PISN at baseline and 6 months were 0.018 (95% CI: 0.001–0.045) and 0.022 (95% CI: 0.002–0.058), respectively, very close to the design assumption of ICC = 0.02, validating the design effect calculation (Supplementary Table 2).

### 3.4 Secondary outcomes: burnout and turnover intention

**Figure 3:**
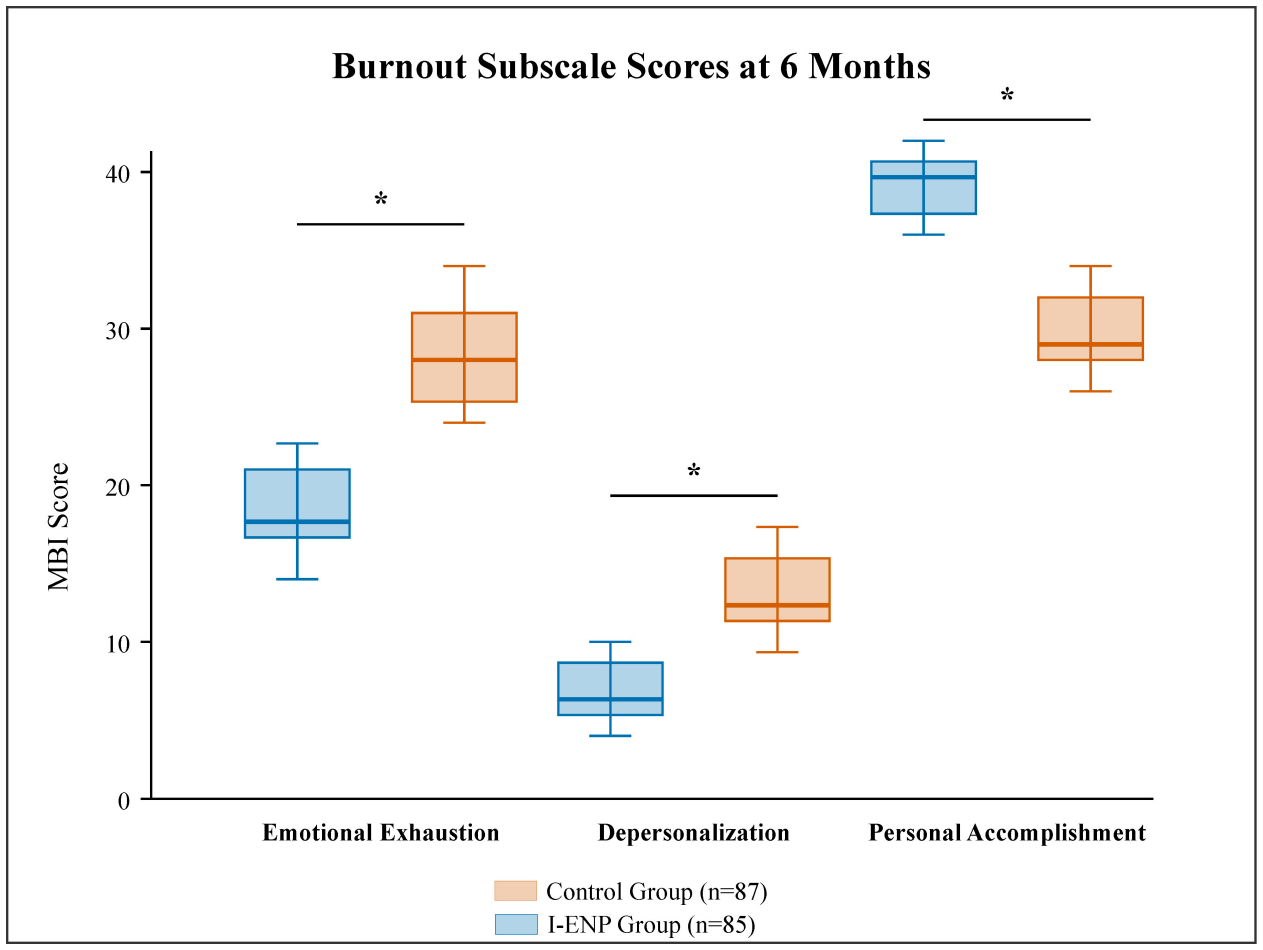
Burnout subscale scores at 6 months post-intervention. Box plots showing scores for emotional exhaustion (EE), depersonalization (DP), and personal accomplishment (PA). The box represents the interquartile range (IQR), the horizontal line inside represents the median, and whiskers extend to 1.5 times the IQR. Between-group comparisons were performed using linear mixed-effects models accounting for clustering *P < 0.001.

Significant improvements were observed for all burnout dimensions in the I-ENP group compared to the control group at 6 months (Table 2 and Figure 3). The I-ENP group experienced a substantial decrease in Emotional Exhaustion scores (adjusted mean difference, −8.2; 95% CI, −10.5 to −5.9; *P* < 0.001) and Depersonalization scores (adjusted mean difference, −4.1; 95% CI, −5.7 to −2.5; *P* < 0.001). Concurrently, their Personal Accomplishment scores significantly increased (adjusted mean difference, 6.5; 95% CI: 4.8–8.2; *P* < 0.001). These positive effects on burnout remained significant at the 9-month follow-up. The observed ICCs for MBI subscales ranged from 0.015 to 0.021 across time points, again validating our design assumption.

At the 6-month assessment, the proportion of nurses reporting a strong intention to leave their job was significantly lower in the I-ENP group (10 of 85, 11.8%) compared to the control group [26 of 87, 29.9%; Odds Ratio (OR), 0.31; 95% CI: 0.14–0.69; *P* = 0.003].

### 3.5 Subgroup analysis

**Figure 4:**
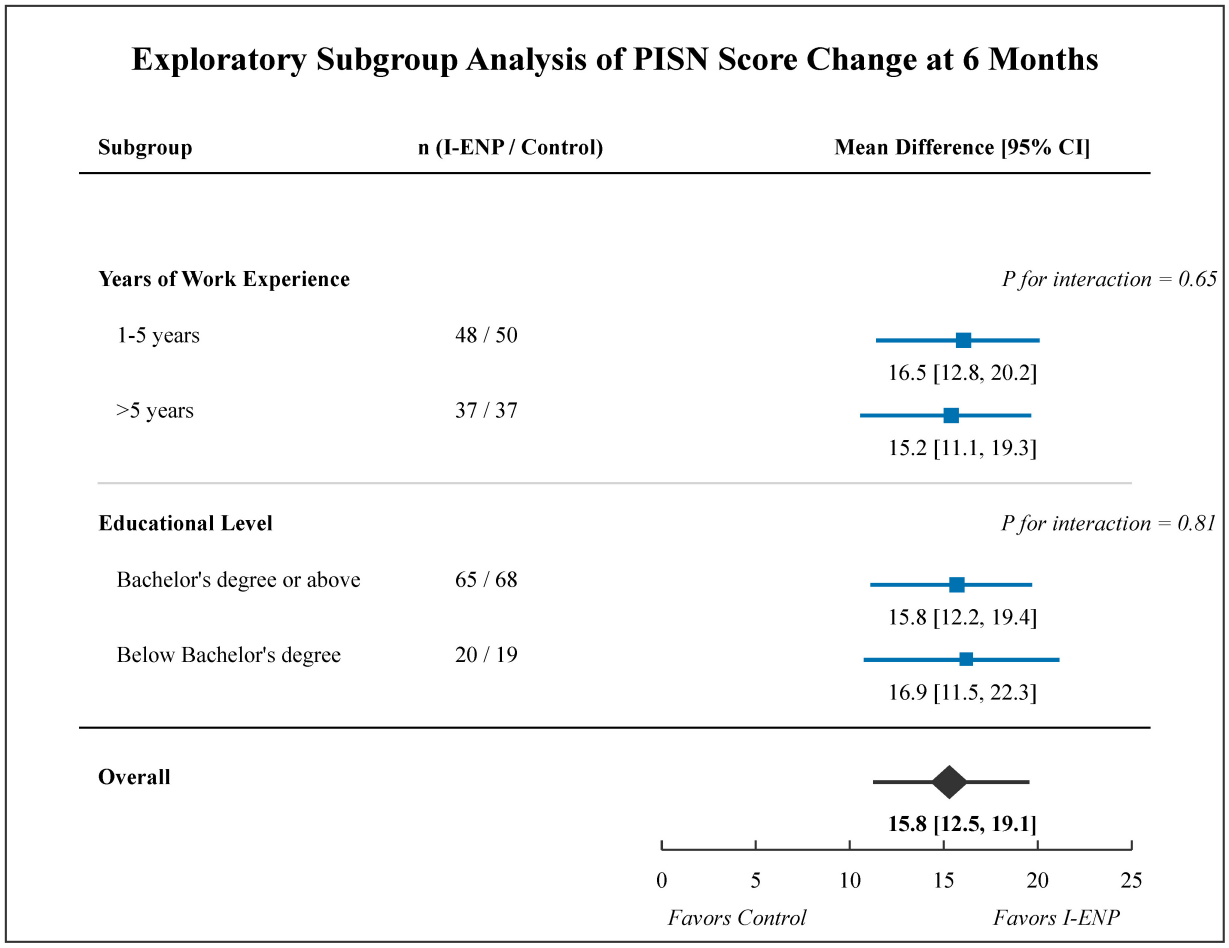
Exploratory subgroup analysis for the primary outcome at 6 months. Forest plot showing the adjusted mean difference in PISN score change (I-ENP minus control) stratified by years of work experience and educational level. Error bars indicate 95% confidence intervals. Analyses are exploratory; *P*-values for interaction indicate whether the intervention effect differed significantly between subgroups.

Subgroup analyses examining the intervention effect on the primary outcome (PISN change at 6 months) stratified by years of nursing experience (1–5 vs. >5 years) and educational level (Bachelor's degree or above vs. below Bachelor's degree) were pre-specified and exploratory in nature. Among nurses with 1–5 years of experience (*n* = 48 intervention, *n* = 50 control), the mean PISN difference at 6 months was 16.5 (95% CI: 12.8–20.2); among nurses with >5 years of experience (*n* = 37 intervention, *n* = 37 control), it was 15.2 (95% CI: 11.1–19.3). For nurses with a Bachelor's degree or above (*n* = 65 intervention, *n* = 68 control), the mean difference was 15.8 (95% CI: 12.2–19.4); for those with education below Bachelor's degree (*n* = 20 intervention, *n* = 19 control), it was 16.9 (95% CI: 11.5–22.3). There was no significant interaction between intervention group and either subgroup variable (*P* for interaction = 0.65 for experience, *P* = 0.81 for education), suggesting broadly consistent effects across experience levels and educational backgrounds (Figure 4).

## 4 Discussion

This cluster randomized controlled trial provides promising evidence that an integrated empowerment and narrative nursing program (I-ENP) may be effective in enhancing professional identity and reducing burnout among neonatal nurses. The findings demonstrate not only statistically significant but also clinically meaningful improvements in the primary and secondary outcomes, which were sustained 3 months after the intervention concluded.

The substantial increase of nearly 16 points in the professional identity score in the intervention group is a key finding. This suggests that the I-ENP successfully addressed core elements that shape a nurse's professional self-concept. The empowerment modules likely contributed by enhancing nurses' clinical competence, communication skills, and sense of control over their work environment, which are known to be foundational to professional pride and confidence (28). The narrative nursing component complemented this by providing a structured space for reflection, allowing nurses to process difficult emotions and reaffirm the values and meaning that initially drew them to the profession (29). This dual approach of equipping nurses with practical skills while also nurturing their inner professional values appears to be a powerful combination for fostering a robust professional identity.

The intervention's impact on all three dimensions of burnout is equally important. The reduction in emotional exhaustion and depersonalization, coupled with an increase in personal accomplishment, indicates a comprehensive alleviation of the burnout syndrome. This aligns with previous studies showing that empowerment interventions can reduce emotional strain by improving coping resources (30), while narrative practices can decrease depersonalization by enhancing empathy and reconnecting nurses with the humanistic aspects of their work (31). Our integrated program effectively targeted both the organizational/skill-based and the existential/emotional aspects of burnout, leading to a more holistic and potent effect than single-focus interventions might achieve.

The significant reduction in turnover intention in the I-ENP group (11.8 vs. 29.9% in control) carries substantial organizational and clinical implications. Nurse turnover is costly—estimated at 0.5–2 times annual salary—and disrupts continuity of care. By reducing turnover intention, the I-ENP may contribute to workforce stability and, indirectly, to the maintenance of clinical expertise and team cohesion in the NICU. This aligns with the conceptual model underlying the intervention: strengthening professional identity and reducing burnout addresses root causes of turnover rather than merely addressing surface-level dissatisfaction.

The subgroup analyses revealed that the intervention was broadly effective across experience levels and educational backgrounds, with no significant interactions. However, these subgroup analyses should be interpreted with caution given the exploratory nature and small subgroup sample sizes; the findings suggest that the I-ENP may be broadly effective across nurse experience levels and education backgrounds, but larger studies with adequate statistical power for interaction testing are needed to definitively assess differential effectiveness.

### 4.1 Limitations

This study has several limitations. First, the trial involved only four hospitals (clusters) randomized in a single Chinese province, which increases vulnerability to baseline imbalance and limits the stability of variance estimations. While our sensitivity analyses (cluster-level *t*-tests and bootstrap resampling) supported the robustness of the main findings, the small number of clusters means conclusions should be interpreted as promising but not definitive, and warrant replication in other settings with more clusters. Our conclusions rest on assumptions inherent in the cluster randomized design, including that cluster allocation was truly random and that there were no unmeasured cluster-level confounders (e.g., differences in hospital culture or leadership style) that could affect outcomes. Additionally, generalizability is limited to tertiary-level NICUs in Hebei Province with similar staffing and organizational structures; the results may differ in resource-limited settings or in NICUs with markedly different demographic or organizational characteristics.

Second, lack of blinding of participants and facilitators, inherent to psycho-educational interventions, could lead to expectancy effects; however, outcome assessors and data analysts were blinded to group assignment, reducing detection bias. Third, reliance on self-reported outcomes (questionnaires) rather than objective measures may be subject to social desirability bias; however, all outcomes were measured using validated, widely-used instruments.

Fourth, an important limitation is that we measured only nurse-level outcomes (professional identity, burnout, and turnover intention) and did not collect neonatal patient outcomes such as adverse events, mortality, family satisfaction, or NICU quality indicators. While improvements in nurse professional identity and reduced burnout are theoretically linked to improved care quality, this link remains hypothetical in our study. Future research should examine whether nurse-focused interventions such as I-ENP translate into measurable improvements in patient safety, clinical outcomes, and family-centered care quality.

Fifth, the trial was not prospectively registered in a clinical trial registry prior to commencement, which is a limitation for transparency and external accountability.

Sixth, the measurement of turnover intention using a single dichotomous item rather than a multi-item scale may lack sensitivity to gradations or ambivalence in turnover intentions and does not capture the strength or urgency of intention. Future studies should use validated multi-item turnover intention scales (e.g., the three-item Turnover Intention Scale) to provide more nuanced assessment of this important outcome.

Seventh, we acknowledge that in a single-city setting with potential for staff rotation or shared training venues, contamination between intervention and control groups was a realistic concern. While we implemented measures to minimize this risk (hospital-level randomization, separate intervention venues, prohibition of material sharing), some indirect contamination through informal networks cannot be completely ruled out, particularly if nurses from different hospitals interacted socially or professionally outside the study setting.

Eighth, multiple related outcomes (four burnout/professional identity dimensions) were examined at two post-intervention time points (6 and 9 months), which modestly increases the possibility of type I error, though the consistency of findings across outcomes and the magnitude of effect sizes suggest conclusions are robust.

## 5 Conclusions

This cluster randomized trial provides promising evidence that an integrated empowerment and narrative nursing program may be effective in enhancing professional identity and alleviating burnout among neonatal nurses, suggesting it is a potentially useful intervention strategy to support nurse wellbeing, reduce turnover intention, and maintain workforce stability. The findings warrant further evaluation in diverse geographic regions and healthcare contexts to determine broader applicability and sustainability.

## Data Availability

The original contributions presented in the study are included in the article/Supplementary material, further inquiries can be directed to the corresponding authors.
