# Supplementary material for: The efficacy of an integrated empowerment and narrative nursing program on enhancing professional identity and alleviating burnout among neonatal nurses: a cluster randomized controlled trial
Source: Front Public Health. 2026 Jan 20;13:1745441. doi: 10.3389/fpubh.2025.1745441 (PMC12864475; doi:10.3389/fpubh.2025.1745441)
Supplement: Supplementary file 1 [file Table_1.docx]

**Supplementary Table 1. Detailed Content of the Integrated Empowerment and Narrative Program (I-ENP)**

| **Module/Session** | **Time** | **Frequency** | **Key Themes and Activities** |
| --- | --- | --- | --- |
| **Part 1: Empowerment Education Modules** | | | |
| Module 1: Professional Competence | Month 1 | 1 × 2-hour workshop | • Review of cutting-edge neonatal care techniques • Case discussion on complex clinical scenarios • Goal setting for professional development |
| Module 2: Stress Management | Month 2 | 1 × 2-hour workshop | • Identifying workplace stressors • Practicing mindfulness and relaxation techniques (e.g., progressive muscle relaxation, guided imagery) • Building emotional regulation skills |
| Module 3: Effective Communication | Month 3 | 1 × 2-hour workshop | • Communicating with distressed families • Assertive communication and conflict resolution with colleagues • Role-playing difficult conversations (e.g., discussing poor prognosis with parents) |
| Module 4: Teamwork & Collaboration | Month 4 | 1 × 2-hour workshop | • Understanding team dynamics and roles • Fostering a culture of psychological safety • Team-based problem-solving exercises |
| **Part 2: Narrative Nursing Sessions** | | | |
| Sessions 1-6 | Months 1-6 | 1 × 1.5-hour session every 2 weeks | • **Story Sharing:** Each session, 2-3 nurses share a significant clinical story (a success, a challenge, a loss) • **Reflective Writing:** Guided prompts for writing "nursing diaries" to explore feelings and meanings • **Group Dialogue:** Facilitated discussion focusing on active listening, empathy, and identifying shared themes • **Peer Support:** Creating a safe space for validation, encouragement, and processing difficult experiences |

**Notes:** I-ENP, Integrated Empowerment and Narrative Program. Sessions were delivered in small groups (8-10 nurses per group) by trained facilitators (a senior nursing manager and a certified psychological counselor). Attendance rates among participants: Mean number of empowerment workshops attended was 3.8 out of 4 (SD = 0.4); mean number of narrative sessions attended was 5.7 out of 6 (SD = 0.5). Overall, 91.8% of participants completed all four empowerment modules, and 94.1% attended at least five of the six narrative sessions.

**Supplementary Table 2. Intracluster Correlation Coefficients (ICCs) for Primary and Secondary Outcomes**

| **Outcome** | **Time Point** | **ICC** | **95% Confidence Interval** |
| --- | --- | --- | --- |
| Professional Identity (PISN) | Baseline (T0) | 0.018 | 0.001–0.045 |
|  | 6 Months (T1) | 0.022 | 0.002–0.058 |
| Emotional Exhaustion (MBI-EE) | Baseline (T0) | 0.015 | 0.001–0.042 |
|  | 6 Months (T1) | 0.019 | 0.001–0.051 |
| Depersonalization (MBI-DP) | Baseline (T0) | 0.017 | 0.001–0.046 |
|  | 6 Months (T1) | 0.021 | 0.002–0.053 |
| Personal Accomplishment (MBI-PA) | Baseline (T0) | 0.016 | 0.001–0.044 |
|  | 6 Months (T1) | 0.019 | 0.002–0.050 |

**Notes:** ICC, Intracluster Correlation Coefficient; PISN, Professional Identity Scale for Nurses; MBI, Maslach Burnout Inventory. ICCs were estimated from the linear mixed-effects models used for primary analyses. The observed ICCs are very close to the design assumption (ICC = 0.02), validating the design effect calculation. ICCs represent the proportion of variance in outcomes attributable to clustering by hospital.

**Supplementary Table 3. Cluster-Level Sensitivity Analysis Results**

| **Outcome** | **Analysis Method** | **Intervention Cluster Mean Change (SD)** | **Control Cluster Mean Change (SD)** | **Mean Difference (95% CI)** | **P-value** |
| --- | --- | --- | --- | --- | --- |
| Professional Identity (PISN) | Cluster-level independent samples t-test | 18.3 (2.1) | 2.4 (1.9) | 15.9 (11.3–17.1) | 0.002 |
|  | Bootstrap resampling (1000 iterations) | 95% Bootstrap CI: [11.8, 17.6] | | | — |

**Notes:** Cluster-level sensitivity analyses compared the mean change in PISN scores from baseline to 6 months between the intervention clusters (n=2 hospitals) and control clusters (n=2 hospitals). The cluster-level independent samples t-test compared the mean of the two intervention cluster changes to the mean of the two control cluster changes. Bootstrap resampling was performed with 1000 iterations at the cluster level to obtain confidence intervals. Results support the robustness of the main individual-level findings.
